# Augmented wound healing potential of photosensitive GelMA hydrogel incorporating antimicrobial peptides and MXene nanoparticles

**DOI:** 10.3389/fbioe.2023.1310349

**Published:** 2023-12-21

**Authors:** Chengzhi Liang, Hongyu Wang, Zhihao Lin, Chengdong Zhang, Guoming Liu, Yanling Hu

**Affiliations:** ^1^ Department of Orthopaedic Surgery, Affiliated Hospital of Qingdao University, Qingdao, Shandong, China; ^2^ Department of Orthopaedic Surgery, The Second Affiliated Hospital of Nanchang University, Jiangxi, China

**Keywords:** composite hydrogel, antimicrobial peptides, MXene nanoparticles, wound dressing, GelMA

## Abstract

**Introduction:** Wound healing is a delicate and complex process influenced by many factors. The treatment of skin wounds commonly involves the use of wound dressings, which remain a routine approach. An ideal dressing can provide protection and a suitable environment for wound surfaces by maintaining moisture and exhibiting good biocompatibility, mechanical strength, and antibacterial properties to promote healing and prevent infection.

**Methods:** We encapsulated tick-derived antibacterial polypeptides (Os) as a model drug within a methylacrylyl gelatin (GelMA) hydrogel containing MXene nanoparticles. The prepared composite hydrogels were evaluated for their wound dressing potential by analyzing surface morphology, mechanical properties, swelling behavior, degradation properties, antibacterial activity, and cytocompatibility.

**Results:** The results demonstrated excellent mechanical strength, swelling performance, degradation behavior, and antibacterial activity of the prepared composite hydrogels, effectively promoting cell growth, adhesion, and expression of antibacterial peptide activity. A full-thickness rat wound model then observed the wound healing process and surface interactions between the composite hydrogels and wounds. The composite hydrogel significantly accelerated wound closure, reduced inflammation, and sped epithelial formation and maturation.

**Discussion:** Incorporating antibacterial peptides into GelMA provides a feasible strategy for developing excellent antibacterial wound dressings capable of tissue repair. In conclusion, this study presents a GelMA-based approach for designing antibacterial dressings with strong tissue regenerative ability.

## Introduction

The skin is the body’s largest organ, which is composed of the cell-protective layer (epidermis and dermis) and its secretions. An individual’s body play a vital role in protecting themselves from various microorganisms that continually attack them (Gurtner et al., 2008; Zhang et al., 2023). The healing of wounds is a complex biological process, which is very important for the recovery of tissue function after injury. There are three stages to the normal wound healing process: (a) inflammation, (b) proliferation (new tissue formation), and (c) remodeling (Gurtner et al., 2008). The three stages may coincide in time but have completely different processes. The healing processes of a wound, many exogenous factors will delay the healing speed of wound healing. When wound healing is stalled by bacterial infections, increased exudation can occur granulation tissue formation at the wound site is inhibited; therefore, wound healing is slowed down (Fu et al., 2021). The market demand for wound dressings that can accelerate wound healing, keep bleeding under control, and prevent infection is huge in skin wound treatment (Fu et al., 2021). It is currently impossible for traditional dressings like gauze to play more than a passive role, such as shielding, moisturizing, absorbing, and exuding, which prevents external stimulation from causing damage, few have antibacterial or activation of endogenous healing factors. For an ideal dressing, the biomaterial must be biocompatible, tissue regenerative, and immune to bacterial infection. Researchers have dedicated their efforts to developing a wide range of wound dressings aimed at promoting skin tissue regeneration. Some of the materials that have garnered attention in this regard include gelatin, chitosan, silk fibroin, and many others.

GelMA was first synthesized by Bulcke et al. through the reaction of gelatin and methacrylic anhydride (MA) in the presence of various photoinitiators. GelMA demonstrates good compatibility and biodegradability with various cell types, thus improving hydrogels’ biological properties (Fan, 2023; do Nascimento et al., 2023). Aubin et al., 2010 cultured several cell types on GelMA hydrogel and showed good cell proliferation. Nikkhah et al., 2012 demonstrated the formation of large vascularization networks in micropatterned GelMA structures. The use of GelMA and its composites in skin wound repair is widespread currently. However, GelMA’s low mechanical strength is not conducive to skin shaping during healing, and alone GelMA has limited antibacterial ability, which limits regenerative stimulation of skin wounds. Different nanoparticles, biopolymers and synthetic nanoparticles are added to the gel to improve its mechanical properties (Xu et al., 2023). Among many loaded nanoparticles, due to its unique structure and properties, MXenes have caught scientists’ attention recently. MXene exhibits numerous distinctive physicochemical properties - for instance, it has inherent hydrophilic functional groups (-OH, -O, etc.) on its surface, eliminating intricate surface modifications typically required for hydrophobic nanoparticles (Huang et al., 2018). Furthermore, MXene demonstrates exceptional metallic conductivity and outstanding biocompatibility, enabling *in vivo* removal and degradation (Liang et al., 2023). These advantageous characteristics have garnered significant attention from nanomedicine researchers, leading to its extensive fabrication use in wound dressings recently.Li et al. utilized the photothermal properties and broad-spectrum antibacterial activity of MXene to prepare MXene@PVA hydrogels. In a mouse wound model, this hydrogel was found to effectively suppress wound infection and promote skin wound healing (wound healing rate of 98%) (Yan Li et al., 2022). Li et al. prepared composite sponges by incorporating MXene-based nanomaterials into a chitosan sponge (CH) network. The composite sponge exhibited excellent antibacterial activity. *In vivo* experiments showed a wound healing rate of 84% on day 9 (Shanshan Li et al., 2022). Zhou et al., 2021a prepared HPEM scaffold through the reaction of polyglycerol-ethylenimine, MXene@PDA nanoparticles and HCHO. It had high antibacterial activity and could stimulate cell proliferation, up-regulating the expression of genes such as α-smooth muscle actin, COL III and VEGF.

Bacterial infection is a key factor affecting skin wound healing. It can increase exudate production at the wound site and prevent granulation tissue formation (Liang et al., 2022). In order to prevent and treat wound infections, antibiotics are widely used. However, improper or excessive use can lead to bacterial resistance and reduced therapeutic efficacy (Chen et al., 2018; Ren et al., 2022). In recent years, antimicrobial peptides like tick-derived Os have demonstrated significant potential for managing wound infections (Cai et al., 2021). Produced by plants, animals, fungi, protozoa and bacteria, antimicrobial peptides are essential immune elements considered much safer than antibiotics (Zhang et al., 2023). Locally applying AMPs encapsulated within various dressings appears to be a good option for treating infected wounds or aiding tissue regeneration. Nanofiber-based wound dressings have a scaffold structure mimicking skin characteristics with a large surface area to volume ratio. This allows for cell attachment and exudate absorption with tunable porosity, facilitating nutrient permeation, gas exchange, and controlled drug release. These properties seem ideal for dressings in wound healing. Incorporating IP-1 into nanocomposite materials (Wei et al., 2020) and W379 with large pores into nanofiber hydrogels (John et al., 2023) enhanced diabetic wound healing and antibacterial activity against wound infection. Additionally, a novel gene delivery system based on AuNPs decorated with AMP (LL37) showed promising effects for local diabetic wound treatment regardless of bacterial infection (Wang et al., 2018).Various antimicrobial peptides have been shown to accelerate *in vivo* wound healing by promoting epithelial re-formation and granulation tissue formation (Wuersching et al., 2021). They also support angiogenesis via the formyl peptide receptor-like 1 pathway, inducing endothelial tube formation (Yang et al., 2020). This upregulates angiogenic proteins and increases vascular endothelial growth factor production, enhancing wound healing by inducing fibroblast differentiation into myofibroblasts. Fibroblast contractility is thereby promoted through enhanced α-smooth muscle actin expression (Sali et al., 2019; Reis Zambom et al., 2020).

Most of the current studies simply use the photothermal properties of MXene nanomaterials to achieve antibacterial effects, while in this study, antimicrobial peptides were added to achieve more efficient synergistic antibacterial effects. At the same time, there are relatively few studies on tick-derived antimicrobial peptides. Therefore, in this study, GelMA hydrogel loaded with antimicrobial peptides and MXene nanoparticles was prepared to explore its role in the healing of infected wounds, aiming to elucidate the biological properties and potential molecular mechanisms of the synthesized gel. We loaded the antibacterial peptides onto GelMA and coated the GelMA with MXene nanoparticles. This confirmed that the prepared photosensitive composite hydrogels had good hydrophilic and antibacterial properties. Then, we verified the good biocompatibility of these nanocomposites through examining cell proliferation. Finally, in the full-thickness defect repair mouse model, we found that the prepared photosensitive composite hydrogel had a good effect on skin wound repair. This is helpful for providing theoretical support for skin tissue engineering.

## Material and methods

### Synthesis of GelMA hydrogel

The dried GelMA was dissolved in phosphate buffer (PBS) and prepared into 5% (w/v) GelMA solution, after adding photoinitiator, the solution was injected into a cylindrical polytetrafluoroethylene (PTFE) mold (d = 10 mm, h = 3 mm) and solidified for 30 s with 10 mW/cm^2^ ultraviolet light (405 nm) to obtain GelMA hydrogel.

### Synthesis of GelMA/Os composite hydrogel

MXene solution (5% w/v) was prepared by the same method as above. After adding photoinitiator, antibacterial polypeptide Os in powder form was added into GelMA solution with Os concentration of 600 μg/mL and ultrasonic dispersion for 10min was made into uniform dispersion solution. The dispersion solution was injected into a PTFE mold and composite hydrogels are prepared by ultraviolet irradiation as described above.

### Synthesis of GelMA/MXene composite hydrogel

The dried GelMA was dissolved in PBS and prepared into GelMA solution with a solubility of 10% (w/v). After adding photoinitiator, the same volume of MXene dispersion solution (200 μg/ml) was mixed into GelMA solution, and ultrasonic dispersion for 10min was made into uniform dispersion solution. Composite hydrogels are prepared as described above.

### Synthesis of GelMA/Os/MXene composite hydrogel

MXene solution (10% w/v) was prepared by the same method as above. After adding photoinitiator, MXene dispersion solution (200 μg/ml) and antimicrobial peptides (600 μg/mL) were added in the same way as above to prepare the mixed solution and ultrasonic dispersing was performed to prepare composite hydrogel.

## Scanning electron microscopy

Four hydrogel samples, GelMA, GelMA/Os, GelMA/MXene, and GelMA/Os/MXene, were selected for SEM analysis. The sample size was about 2 mm^*^2 mm, in a thin film shape, and the surface was sprayed with gold. The surface morphology and pore structure were observed by scanning electron microscope.

### Mechanical properties

Samples of GelMA, GelMA/Os, GelMA/MXene, and GelMA/Os/MXene were selected. The mechanical properties of different hydrogels were tested on a universal test machine (Instron; 5967). The hydrogel sample was made into a cylinder with a diameter of 10 mm and a height of 3 mm. The universal machine tests the strain of four hydrogel samples at a constant speed until the samples are completely broken; Meanwhile, tensile stress values were recorded successively. Draw a hydrogel stress-strain curve.

P=F/S where F is the compression load and S is the cross-sectional area.

## Swelling property

GelMA, GelMA/Os, GelMA/MXene, and GelMA/Os/MXene samples (d = 10 mm, h = 3 mm) were selected, and the original mass Wd of the hydrogel was recorded by weighing with an electronic balance. The hydrogel sample was put into PBS solution at 37°C and samples were immersed. The sample was taken out at the set time point (2 h, 4 h, 6 h, 12 h, 24 h, 36 h) and the mass of the sample (Ws) was recorded to draw the hydrogel swelling curve.

$$\text{Material swelling SR}=(\left(\text{Ws}-\text{Wd})/\text{Wd}\right)\;*\;100$$

### Degradation performance

GelMA, GelMA/Os, GelMA/MXene, and GelMA/Os/MXene samples (d = 10 mm, h = 3 mm) were selected. The initial weight (Mo) of the hydrogel was recorded after 24 h immersion at 37°C PBS to achieve swelling equilibrium. The hydrogel was put into type II collagenase solution (2U/ml), and the hydrogel was taken out at a predetermined time point (2d, 4d, 6d, 8d, 10d, 12d, 14d). The hydrogel was washed with PBS without collagenase, weighed, and recorded as Mi, and the hydrogel degradation curve was drawn.

$$\text{Material degradation rate}=\left(\left(\text{Mo}-\text{Mi}\right)\;/\text{Mo}\right)\;*\;100$$

### Hemolytic properties

The hemolytic properties of the hydrogels were analyzed. Fresh blood (4 mL) was collected from SD rats into anticoagulant tubes and diluted with normal saline (5 mL) for later use. Diluted blood was mixed with normal saline as a negative control (0% lysis) and mixed with water as a positive control (100% lysis). Three groups of different hydrogels prepared in the experimental group were transferred to a centrifuge tube containing 1 mL normal saline, 20 μL blood was added, and the test samples of the above groups were incubated at 37°C for 1 h. The samples were then centrifuged at 2,000 rpm for 5 min, and the absorbance of the supernatant was measured at 540 nm. Each sample was prepared in triplicate.

$$\text{Hemolysis Rate }\left(\text{HR}\right):\text{HR}=\left[\;\left(\text{ODt}-\text{OD}0\right)\;/\;\left(\text{ODp}-\text{OD}0\right)\;\right]*\;100\%$$

ODt is the absorbance value of experimental group, ODp is the absorbance value of positive control group, and OD0 is the absorbance value of negative control group.

### Antibacterial activity

*Staphylococcus aureus* and *Escherichia coli* were used to detect the antibacterial activity of hydrogels in each group. The hydrogel samples were made into 10 mm cylinders under ultraviolet light and used on an ultra-clean platform. The two bacteria (4.0 × 10^4^ mL^−1^) were cultured in lysozyme broth (LB) medium at 37°C for 2 h. Then 200 μL bacterial suspension was added to the surface of the sample and cultured in an incubator at 37°C for 10 h. Finally, the absorbance of bacteria in each group was measured by spectrophotometer at 600 nm, and the antibacterial activity of hydrogel materials in each group was evaluated.

Live/dead experiments were used to investigate the bacteriostatic properties of the hydrogels in each group, and the changes of bacteria on the hydrogels in each group were observed. Different hydrogels were co-cultured with bacterial suspension at 37°C for 10 h. PI and calxanthin were used for bacterial staining. Finally, fluorescence microscopy (TE2000-U; Nikon) look at different samples.

### Cell proliferation experiment

Cell proliferation on the GelMA, GelMA/Os, GelMA/MXene, and GelMA/Os/MXene composite hydrogel was assessed. The mixed solution of each group was injected into 96-well plate (50 micro/well) and irradiated with 405 nm ultraviolet light source to gelate. L929 fibroblasts were inoculated into 96-well plates and incubated for 24 h in a constant temperature incubator at 37°C and 5%CO_2_. At each specific time point (1d, 4d, 7d), the effects of different materials on cell proliferation were assessed using the CCK-8 method. Briefly, the culture medium was replaced with Cell Counting Kit-8 (CCK-8) reagent (CCK-8, Japan). Incubate at 37°C for 2 h. Finally, 100 μL medium was transferred to a new 96-well plate. Subsequently, the absorbance was measured at 570 nm using an enzyme-labeled instrument. The proliferation trend of fibroblasts was mapped.

In the same way as above, the bacteria were stained with Calcein/PI on day 4 and finally observed with a fluorescence microscope.

For cell adhesion experiments using NH3T3 cells, we cultured the cells on different hydrogel. The cultured cells were fixed with 4% paraformaldehyde and washed 3 times with PBS. Cytoskeleton and nucleus staining were performed by FITC and DAPI, respectively. Finally, morphology was observed under cell fluorescence microscope.

### *In vivo* wound healing assessment

Bioactivity analysis of different materials was conducted using adult female rats, aged 10–12 weeks and weighing 200–250 g. All animal experiments were carried out under the approval of Animal Ethics Committee of Affiliated Hospital of Qingdao University. The rats were anesthetized with 2% sodium pentobarbital (40 mg/kg, intraperitoneally injected), and their backs were removed. The round full-layer skin wound model of the rat back was made with eye scissors, and the diameter of the wound was 10 mm. The skin wounds of the rats were treated with different hydrogels, including GelMA, GelMA/Os, GelMA/MXene, and GelMA/Os/MXene. The wound dressings were replaced every 2 days. The experimental animals were housed individually in standard environmental conditions at a temperature of 22°C. The experimental rats were fed in Animal Laboratory Center of Qingdao University with conventional pellet feed and water. All rats were randomly divided into 5 groups with 3 rats in each group. Wound photos were recorded at day 0, 5, 9, and 12 after surgery, and the wound area was measured using ImageJ software. The calculation formula is as follows:

$$\text{Wound closure rate}=\left[\left(A_{0}-A_{t}\right)/A_{0}\right]\times \;100\%$$

In the formula, A_0_ represents the initial area of the wound (t = 0), and A_t_ represents the area of the wound during measurement.

### Histopathological staining

The rats in each group were examined histologically and sacrificed on the 14th day after operation (Cervical dislocation under anesthesia was performed as a strategy for euthanasia). The skin tissue surrounding the wound was excised, fixed in formalin, dehydrated in alcohol, cleared in xylene, embedded in paraffin, and sectioned vertically to a thickness of about 5 mm. Histological changes were observed under light microscopy after HE and Masson staining. In addition, the collagen deposition in the wound was analyzed by Sirius red staining, and the ratio of type ⅰ collagen to type ⅲ collagen was quantitatively analyzed under a polarized light microscope. Major organs (heart, liver, spleen, lung, and kidney) of rats in each group were collected for H&E staining to observe the biological toxicity of hydrogel in each group.

### Statistical analysis

Data analysis was performed using Origin 8.0 software (Origin Lab, Los Angeles, CA, United States). The statistical differences between the data were evaluated using one-way analysis of variance (ANOVA) followed by *post hoc* tests. The results are presented as the mean ± standard deviation (SD). Statistical significance was considered when the *p*-value was less than 0.05 (*p* < 0.05).

## Result and discussion

**FIGURE 1:**
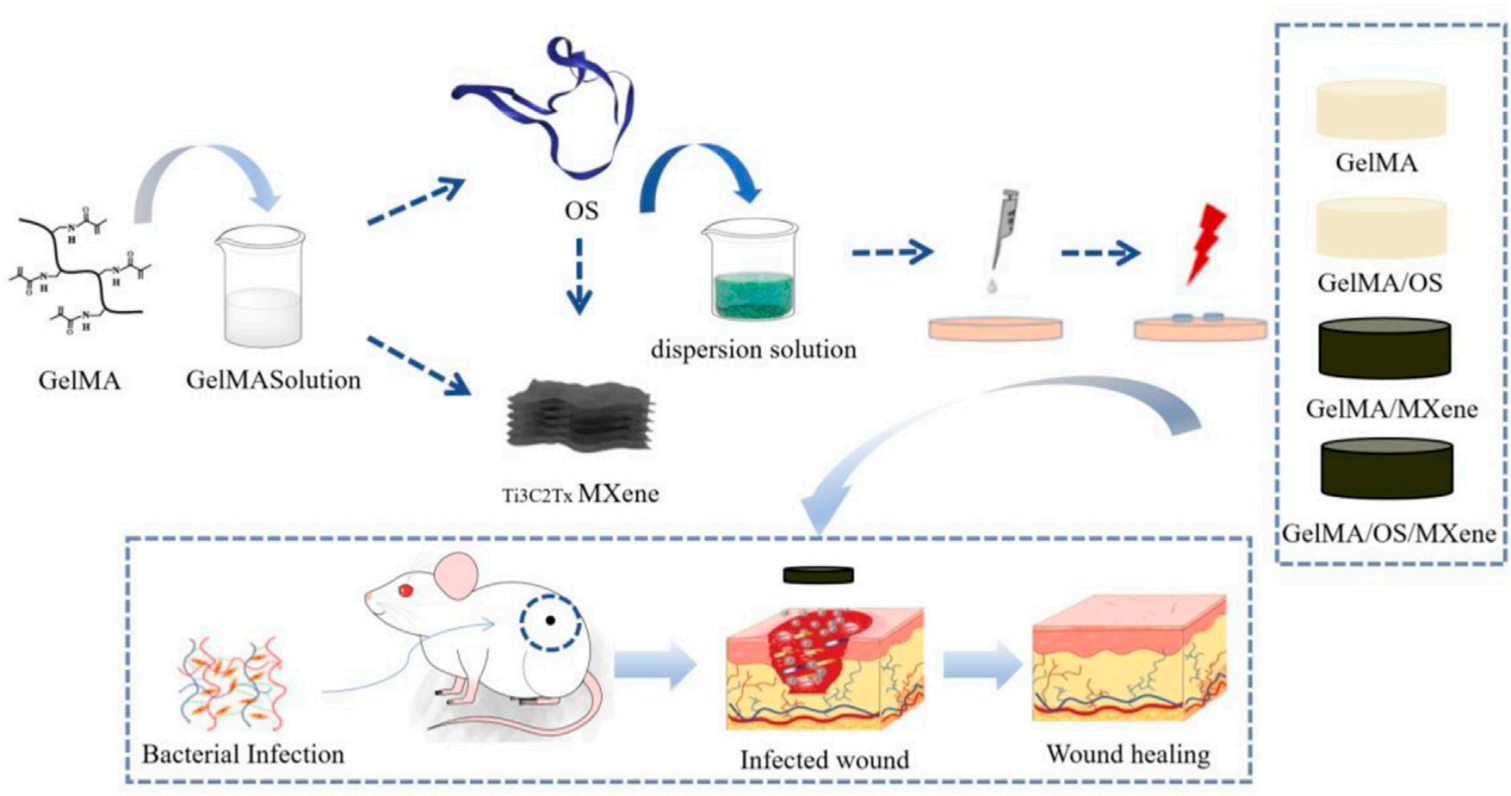
Schematic diagram of the experimental procedure.

As shown in Figure 1, the hydrogel was prepared for performance characterization, antibacterial property verification, *in vitro* cell experiments and animal experiments to explore its effect on wound healing.

### Characterization of composite hydrogel

**FIGURE 2:**
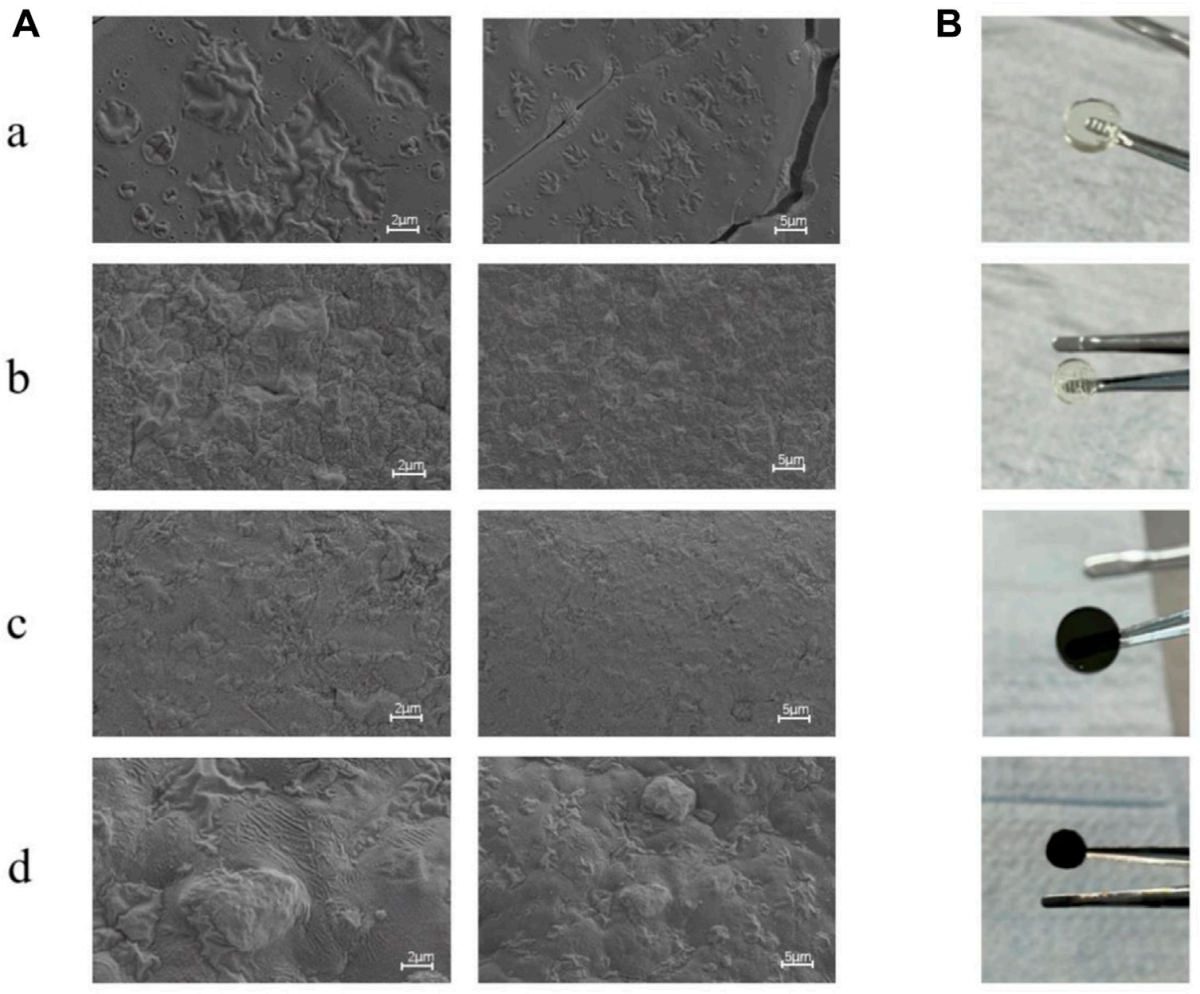
**(A)** SEM image and **(B)** Macroscopic observation image of MXene(a), GelMA/Os(b), Gel/MXene(c), and GelMA/Os/MXene(d) hydrogels. Scale bar lengths are 2 μm and 5 μm.

It is well known that the surface morphology of wound dressings can affect cell adhesion and proliferation, which is an important part of tissue engineering applications. Figure 2 presents the scanning electron microscopy (SEM) images, illustrating the surface morphologies of various gel samples (GelMA, GelMA/Os, Gel/MXene, and GelMA/Os/MXene). It is obvious that the surfaces of pure GelMA in the four groups are smooth and flat (Figures 2A,B). Although the GelMA/MXene gel surface was rougher than the GelMA group (Figures 2E,F), it was not as obvious as the GelMA/Os group (Figures 2C,D), indicating that the addition of MXene nanoparticles had a less obvious effect on the surface morphology of GelMA gel than the antibacterial peptide Os. This may be because GelMA surface functional groups are attached to the ends of polypeptide chains. With the addition of Os and MXene nanoparticles, the surface roughness of the composite gel increased significantly (Figures 2G,H), and some agglomerated particles and hilly undulations appeared on the surface, which may be caused by nanoparticle agglomeration.

### Mechanical properties

**FIGURE 3:**
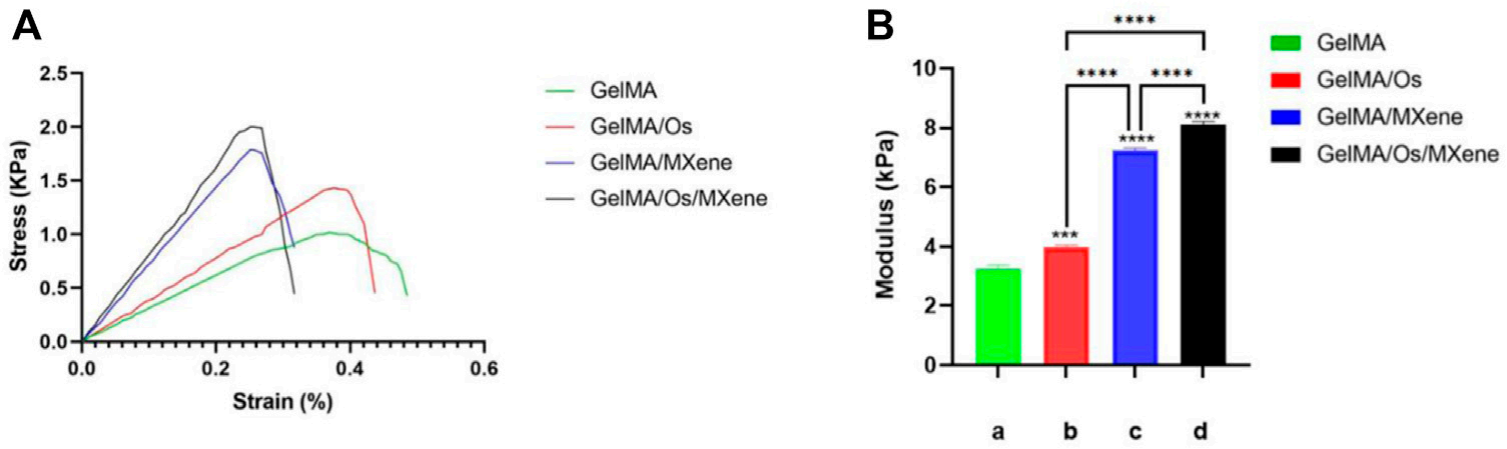
**(A)** Hydrogel strain-pressure curves of different hydrogels, and **(B)** compression modulus of MXene(a), GelMA/Os(b), Gel/MXene(c), and GelMA/Os/MXene(d) hydrogels. *p* < 0.05 (*), *p* < 0.01 (**), *p* < 0.005 (***), *n* = 3.

Similarly, the mechanical properties of wound dressings are also key factors affecting wound healing. Dressings need to have sufficient mechanical strength to accommodate deformation caused by wound contraction. Studies have shown that good mechanical properties are not only beneficial to cell proliferation and adhesion, but also affect cell growth and differentiation (Malan et al., 2016; Reis Zambom et al., 2020). We examined the compressive strength of four groups of hydrogels to verify the effect of MXene and Os on the mechanical properties of GelMA hydrogels. In this experiment, as shown in Figure 3A, the strain-pressure curves of the four groups of hydrogels showed that the maximum compressive strength of GelMA, GelMA/Os, GelMA/MXene, and GelMA/Os/MXene hydrogels was 1.020 ± 0.04 KPa, respectively. 1.433 ± 0.05 KPa; 1.786 ± 0.05 KPa; 2.054 ± 0.07 KPa. Figure 3B shows the compression modulus statistics of the four groups of hydrogels. The compression modulus of the composite hydrogels increased significantly after the addition of Os and MXene, with statistical difference (*p* < 0.05). Previous studies have found that MXene nanoparticles have strong mechanical strength and can improve the compression properties of composite hydrogels. On the other hand, Os itself has abundant functional groups (He et al., 2021), which can be grafted with active groups of MXene or GelMA to engender stronger mechanical properties of composite hydrogels.

### Swelling and degradation properties of composite hydrogel

**FIGURE 4:**
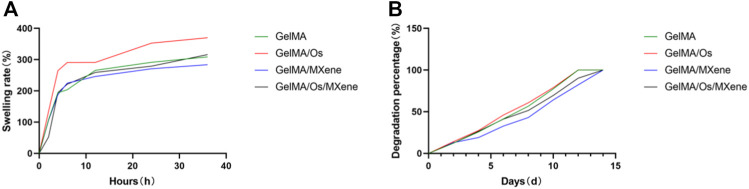
**(A)** The swelling curve and **(B)** degradation curve of MXene, GelMA/Os, Gel/MXene, and GelMA/Os/MXene hydrogels.

Hydrogel wound dressing should have good swelling properties, conducive to the removal of wound exudate, and create a moist environment conducive to wound healing. The swelling property of hydrogel was evaluated by studying the swelling rate of the material. As shown in Figure 4, in the first 6 h, the four groups of hydrogels rapidly absorbed water swelled, and increased their mass, and then the rate of increase slowed down gradually but still increased. At about 24 h, the water absorption of the hydrogels reached saturation and were in a swelling equilibrium state, with swelling rate between 280% and 380%. All hydrogels showed good swelling capacity. MXene nanoparticles all contain a large number of hydrophilic groups and show strong hydrophilicity (Rasool et al., 2016; Jin et al., 2021; Guo et al., 2022; Cooksley et al., 2023), but the equilibrium swelling rate of GelMA/MXene hydrogels after the addition of MXene is lower than that of GelMA hydrogels. Our analysis suggests that this may be because the composite hydrogels formed by the two have smaller pore sizes and denser composite structures, leading to a decrease in swelling rate. When MXene and Os were added at the same time, the swelling rate of hydrogel was balanced and compensated, so GelMA/Os/MXene group and GelMA group had similar swelling rates.

Hydrogels should be biodegradable as well as swollen so that they can self-degrade in the process of wound healing to avoid hindering wound contraction and thus affecting wound healing. In this study, as shown in the hydrogel degradation curve, all hydrogel samples were degraded within 14 days, and the degradation rate showed no statistical difference. The results showed that GelMA, Os, and MXene had biodegradability. Probably due to the introduction of MXene nanoparticles, the hydrogel structure became dense, the mechanical strength increased, and the degradation rate was relatively slower than that of the two groups without MXene nanoparticles.

### Hemolytic properties

**FIGURE 5:**
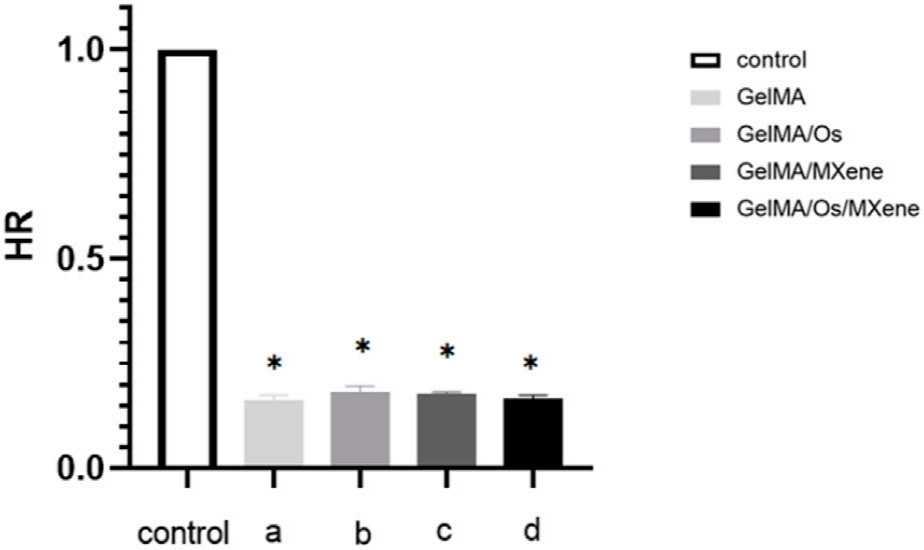
Statistical diagram of hydrogel hemolytic rate. *p* < 0.05 (*), *n* = 3.

When exogenous materials come into direct contact with blood, red blood cells in the blood will rupture, leading to abnormalities and inducing severe coagulation reactions. The lower the hemolytic rate of a certain material, the less likely it is to damage red blood cells and the more suitable it is for materials that come into direct contact with blood. Figure 5 shows a composite hydrogel solution experiment. After statistical analysis, the hemolytic rate of the hydrogel was less than 2% as stipulated by international standards, indicating that the hydrogel has good blood compatibility.

### Antibacterial activity

**FIGURE 6:**
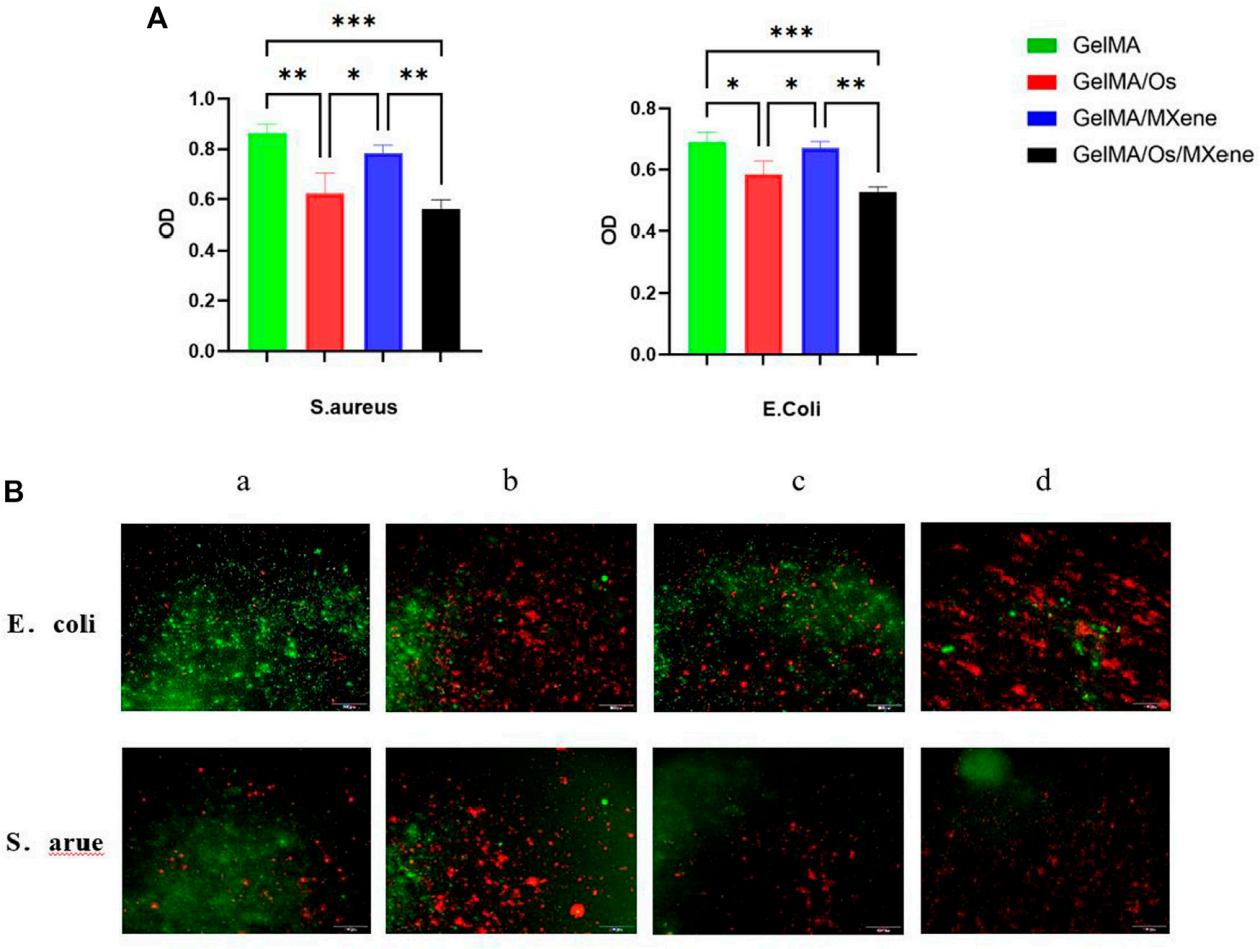
**(A)** Different hydrogels’ antibacterial effectiveness, and **(B)** After treatment with PI and calcein, fluorescence micrographs of *S. aureus* and *E. coli* were obtained. MXene(a), GelMA/Os(b), Gel/MXene(c), and GelMA/Os/MXene hydrogels(d). Scale bar lengths are 100 μm, *p* < 0.05 (*), *p* < 0.01 (**), *p* < 0.005 (***), *n* = 3.

Preventing skin wound infection is of utmost importance in the treatment of skin wounds. Therefore, whether the wound dressing has excellent antibacterial ability becomes an important index to evaluate the dressing. Overuse of antibiotics is one of the challenges in the treatment process, which can cause bacteria to develop resistance to antibiotics and eventually develop chronic infections. In this study, we prepared a new antibacterial dressing by loading antibacterial peptides and nanoparticles MXene. The antibacterial activity of different hydrogels was studied using *E. coli* and *S. aureus*. As shown in the figure, the GelMA/Os hydrogel exhibited better antibacterial activity against *S. aureus E. coli* than the pure GelMA hydrogel, indicating that the antibacterial properties of the antibacterial peptides could be fully displayed in GelMA hydrogel after the combination of antibacterial peptides, and MXene could produce synergic antibacterial effect with the antibacterial peptides after being cross-linked to the composite hydrogel. The OD value is further reduced. As shown in Figure 6A, GelMA/Os/MXene nanocomposite hydrogels showed the highest bacterial inhibition among all the composite hydrogels. Previous studies have shown that MXene nanoparticles themselves have antibacterial properties (Jastrzębska et al., 2017; Shamsabadi et al., 2018). Shamsabadi et al. have observed the destruction of bacterial cells and the leakage of bacterial DNA in the dark environment after eliminating the antibacterial generated by the photothermal effect (Ahmad et al., 2021). To enhance the observation of the antibacterial activity of various composite hydrogels, the bacteria present on the surface of different samples were stained using propidium iodide (PI) and calcein. When observed under a fluorescence microscope, dead bacteria emit a red fluorescence, whereas live bacteria emit a green fluorescence. As depicted in Figure 6B, the pure GelMA gel exhibited the strongest green fluorescence for both *E. coli* and *S. aureus* among all the samples. The gradual enhancement of the red fluorescence region was observed with the addition of Os and MXene. Among the groups, GelMA/Os/MXene exhibited the strongest red fluorescence when compared to the other groups.

### Cell proliferation experiment

**FIGURE 7:**
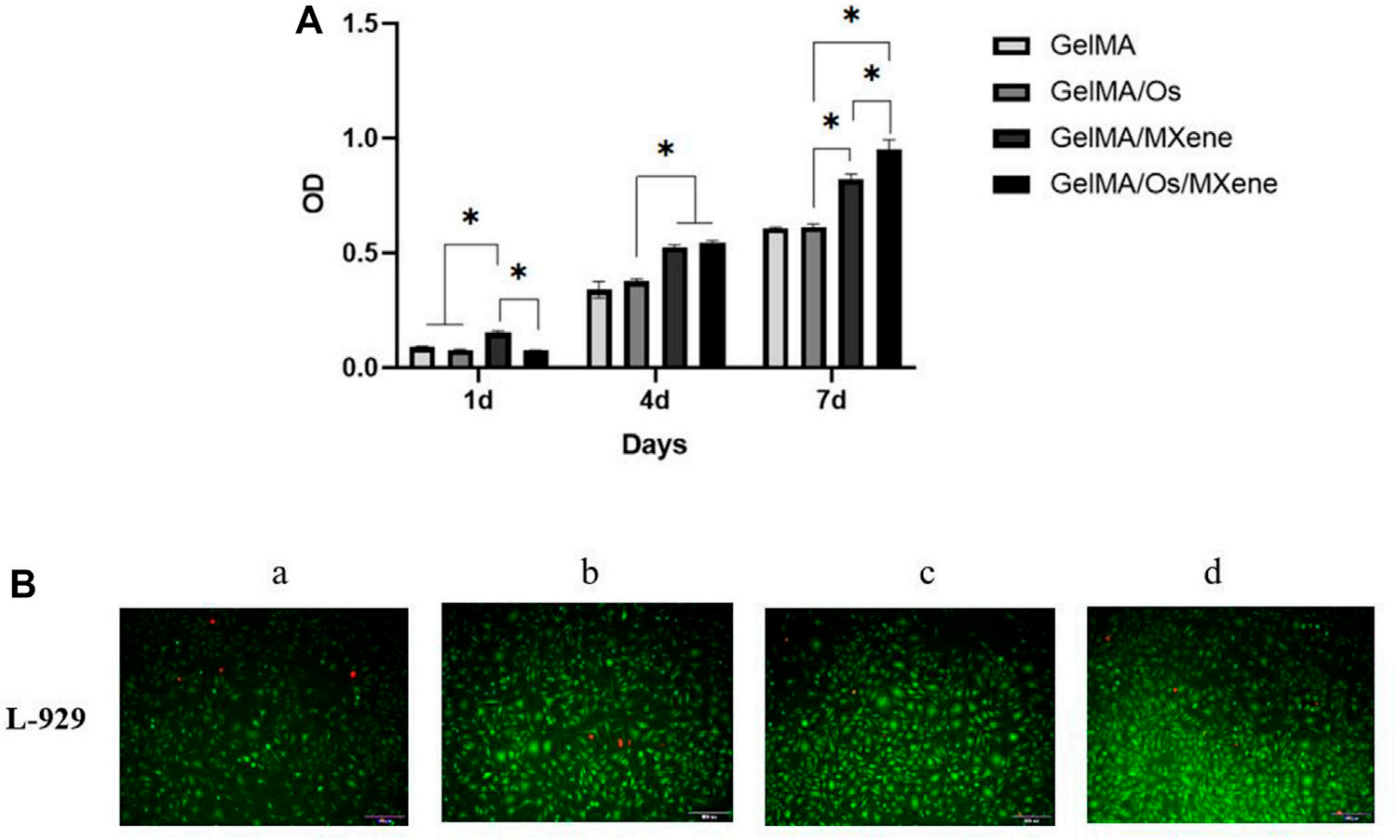
**(A)** Cell proliferation, and **(B)** morphology of L-929 cells cultured on MXene **(a)**, GelMA/Os **(b)**, Gel/MXene **(c)**, and GelMA/Os/MXene hydrogels **(d)**. Scale bar lengths are 100 μm, *p* < 0.05 (*), *n* = 3.

The effects of various compound hydrogels on cell proliferation were investigated using the L929 cell system. The cell proliferation on the surface of different hydrogels was assessed using the CCK-8 method and live/dead staining. As can be seen from Figure 7, the number of L929 cells gradually increased with the extension of culture time. Significantly higher cell numbers were observed in the GelMA/MXene group compared to the GelMA group on the 4th and 7th day of culture, indicating that GelMA/MXene composite hydrogels loaded with Mxene nanoparticles promoted cell proliferation, while GelMA/Os composite hydrogels added with antibacterial peptides had no obvious effect on cell proliferation. GelMA/Os/MXene composite hydrogel group showed the strongest proliferating properties, which may be because the hydrogel hydrophilicity and surface roughness were improved after the addition of MXene and antimicrobial peptides, thus affecting cell survival and adhesion conditions and promoting cell proliferation. Previous studies have suggested that there are abundant hydrophilic functional groups (Anasori et al., 2017; Li et al., 2022)on the surface of MXene, which play an important role in the interaction between materials and cells after being negatively loaded into the composite hydrogel.

### *In vivo* wound healing assessment

**FIGURE 8:**
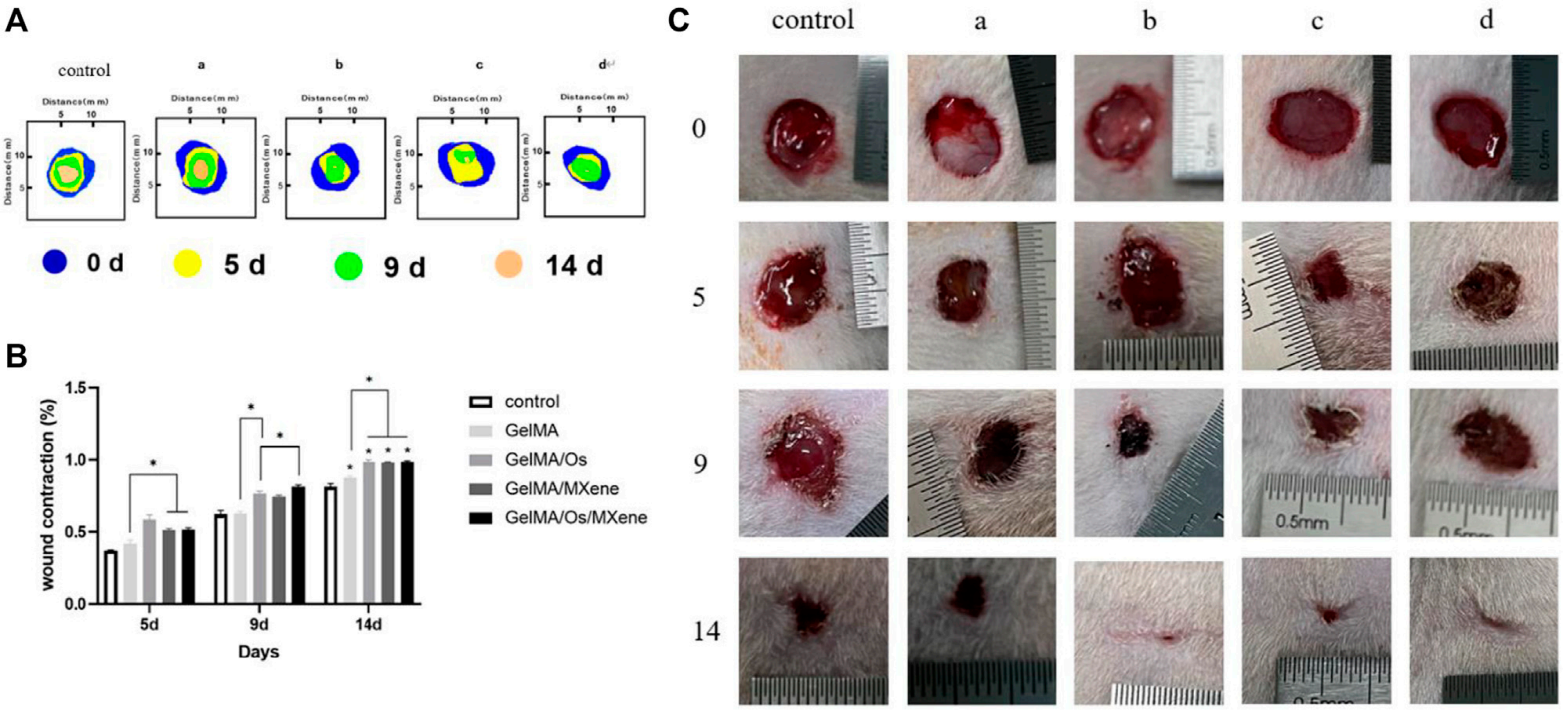
**(A)** The diagram illustrates the progression of wound closure at various time intervals; and **(B)** quantitative statistical analysis of wounds closure; **(C)** Photographs of the wound closure for MXene(a), GelMA/Os(b), Gel/MXene(c), and GelMA/Os/MXene hydrogels(d). *p* < 0.05 (*), *n* = 3.

The skin is the main barrier that protects the body from external irritation and damage. Skin wound healing is a precise and complex process involving a variety of cells, factors, and extracellular components. As shown in Figure 8, the size of wounds in each group tended to decrease with the extension of time, and the untreated control group was the group with the slowest healing speed. Furthermore, the GelMA/Os group exhibited a significantly higher wound healing rate compared to the GelMA group, indicating that adding antimicrobial peptides could improve the repair efficiency of infected wounds. The GelMA/Os/MXene group demonstrated the most rapid reduction in wound area and exhibited the most effective wound closure compared to the other groups. The wound healing rate in the GelMA/Os, GelMA/MXene, and GelMA/Os/MXene groups was significantly higher than that in the GelMA and control groups after 9 days. After 14 days of treatment, wounds in the GelMA/Os, GelMA/MXene, and GelMA/Os/MXene groups were almost completely closed, reaching 98.8%, 98.2%, and 99.1%, respectively. Therefore, in the mouse wound healing model, GelMA/Os/MXene composite hydrogel can significantly accelerate wound healing and shorten wound healing time. It has been reported that MXene nanoparticles have a positive effect on human wound healing and promotes the process of skin healing by affecting the properties of keratinocytes (Zhou et al., 2021b; Zheng et al., 2021). Some studies have applied MXene nanoparticles in medical research and obtained exciting results such as antibacterial, conductive, promoting cell proliferation, and promoting angiogenesis (Zhao et al., 2016; Zhu et al., 2020). Previous studies have highlighted the favorable biocompatibility of GelMA, which supports the growth, differentiation, and stratification of cells, enabling the transformation of keratinocytes into functional multilayer epidermal tissues (Rehman et al., 2019). GelMA’s abundance of functional groups also facilitates interactions with cells, thereby improving cell viability (Gehring et al., 2016).

### Histopathological staining

In order to study inflammation and lesions in tissues or organs, immunohistochemical observation is typically required. Immunohistochemistry is performed by embedding pathological tissues to be observed in paraffin and making paraffin sections. After staining, the presence and location of antigens and inflammatory tissues in cells can be observed.To evaluate the effect of different composite hydrogels on wound healing, the results were assessed by hematoxylin and eosin staining. Hematoxylin-eosin staining produced clear color contrast between the nucleus (blue) and cytoplasm, muscle fibers, collagen fibers, and erythrocytes (varying shades of red). Epidermal healing is equally complex and precise, with the epidermis gradually thickening as cells proliferate during healing. As healing continued, the epidermis gradually became thinner and eventually returned to normal.

**FIGURE 9:**
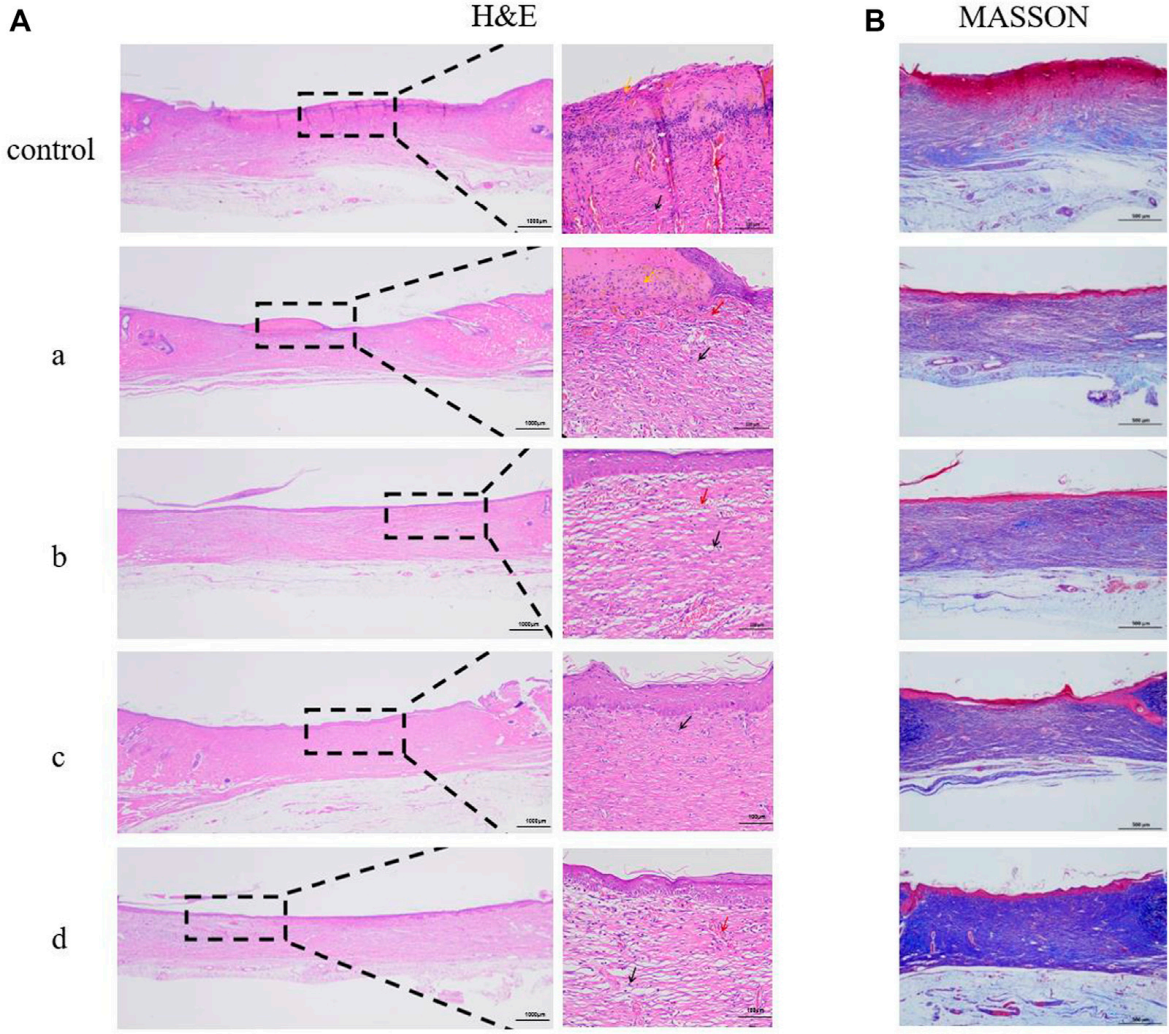
Histological appearance of wounds harvested on days 14 of each group, MXene **(a)**, GelMA/Os **(b)**, Gel/MXene **(c)**, and GelMA/Os/MXene hydrogels **(d)**. **(A)** HE and **(B)** MASSON.

As shown in Figure 9A, the control group without treatment had significantly thicker epidermis than the composite hydrogel group. With the addition of antimicrobial peptides Os and MXene nanoparticles, epidermal thickness decreased in samples, with GelMA/Os/MXene having the thinnest epidermis among groups. The degree of wound healing in GelMA/Os/MXene was higher than other groups.Figure 9B displays collagen deposition results on day 14 via MASSON staining. Collagen deposition at wound sites was significantly higher in GelMA/Os, GelMA/MXene, and GelMA/Os/MXene *versus* GelMA and control groups. Collagen fibers in control and GelMA dermis were irregularly and sparingly arranged, while fibers in GelMA/Os, GelMA/MXene, and GelMA/Os/MXene groups were regularly arranged, indicating more mature regenerated tissue.

**FIGURE 10:**
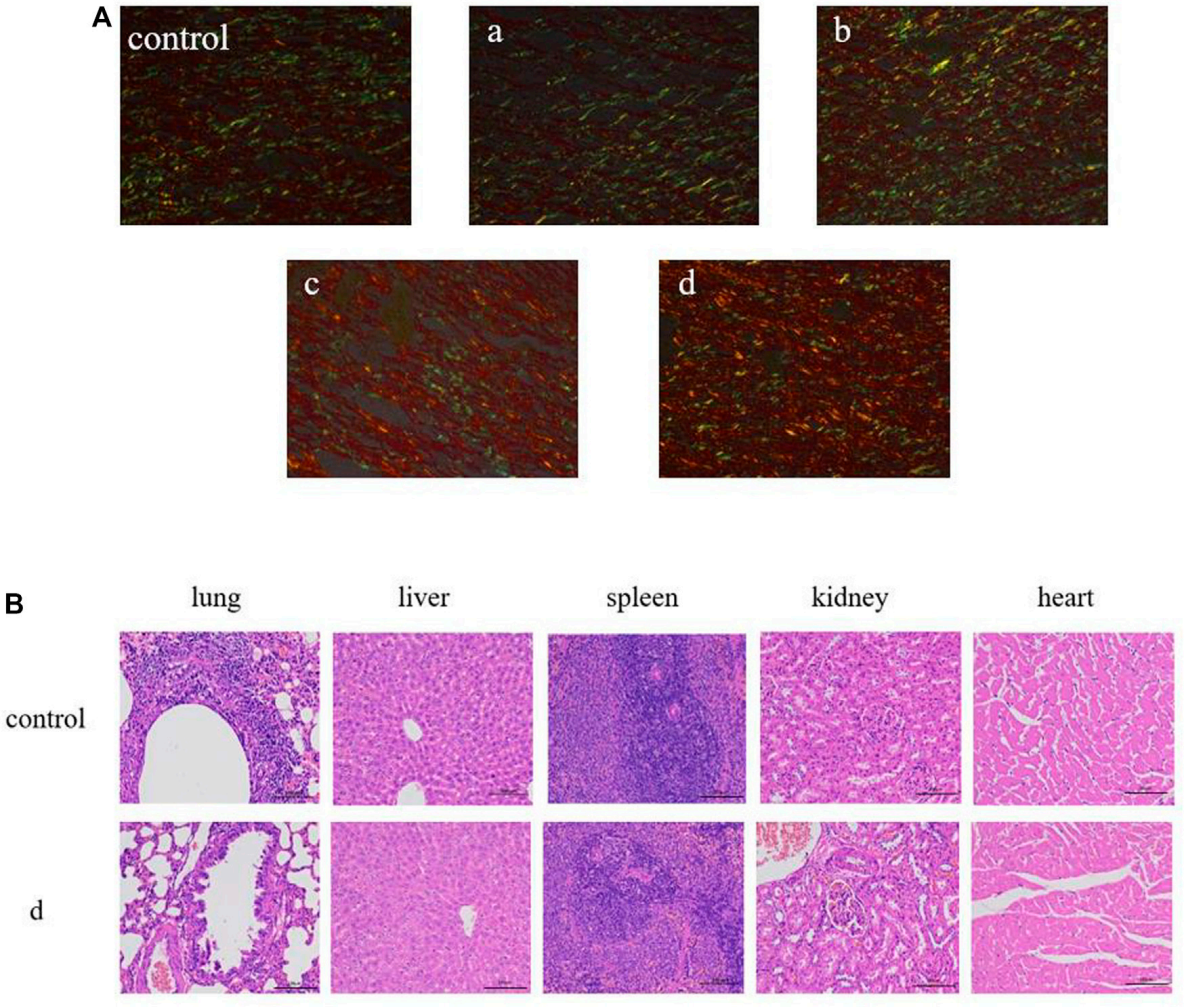
**(A)** Sirius red staining in the wound-healing region at 14 days after MXene (a), GelMA/Os (b), Gel/MXene (c), and GelMA/Os/MXene hydrogels (d) treatments, Magnification, ×200. **(B)** Histological appearance of organ. scale bar lengths are 100 μm.

Collagen plays a vital role in skin tissue, particularly during the process of wound healing. In the early stages of wound healing, the predominant collagen type is thin and loose type III collagen. As tissue repair progresses, there is a gradual transition of the extracellular matrix from type III collagen to type I collagen. This transition is essential in enhancing the tensile strength of the scar tissue (Brice and Diamond, 2020a). Hence, the ratio of type I collagen (COL-I) to type III collagen (COL-III) can serve as an indicator to assess the extent of wound healing. The analysis of collagen types in the wounds was performed using Sirius red staining, where COL-I appeared as orange to red and COL-III appeared as green. As depicted in Figure 10A, the majority of wound collagen in the composite hydrogel group consisted of type I collagen, while the control group exhibited a higher proportion of COL-III, indicating ongoing wound healing. The GelMA group demonstrated increased expression of COL-I compared to the control group, suggesting that GelMA can stimulate wound healing by promoting collagen synthesis (do Nascimento et al., 2023; Rehman et al., 2019). After addition of antimicrobial peptide Os and MXene nanoparticles, the intensity of red fluorescence in skin wound samples increased. Antimicrobial peptides exhibit robust antibacterial activity and effectively inhibit the growth of pathogenic bacteria (Brice and Diamond, 2020b; Rodriguez et al., 2020). Their incorporation into composite hydrogels enhances the antibacterial properties, thereby creating a favorable environment for accelerated wound healing. Additionally, MXene nanoparticles contribute to the enhancement of physical properties in composite hydrogels. These nanoparticles improve mechanical strength, hydrophilicity, and protein adsorption, further enhancing the overall performance of the hydrogel dressing. Furthermore, our findings revealed that the GelMA/Os/MXene group exhibited the highest ratio of type I to type III collagen compared to all other groups. This indicates that the GelMA/Os/MXene group had progressed to the remodeling stage of wound healing and exhibited the highest efficiency in wound repair. In addition, the major organs (heart, liver, spleen, lung, and kidney) of rats in the control group and GelMA/Os/MXenen hydrogel group were observed by HE staining, as shown in Figure 10B. No inflammatory cell infiltration was observed in the major organs of each group, no obvious histological changes occurred, and no experimental animals died *in vivo*. GelMA/Os/MXenen composite hydrogel has good biocompatibility and biosafety.

Hydrogel is a highly beneficial material for wound repair as it can serve as a moist wound dressing, effectively promoting wound healing. It possesses outstanding biocompatibility and degradability, facilitating its interaction with the wound environment. Hydrogel also promotes fibroblast migration, a crucial step in wound healing, and facilitates collagen deposition, leading to the formation of new tissue at the wound site. However, due to the enzymatic degradation of type I and type II collagenase (MMP-1 and MMP-8), hydrogels are easily degraded *in vivo*, so scholars synthesized GelMA hydrogels through the reaction of hydrogels and methacrylic anhydride (MA) in the presence of different photoinitiators to improve the stability of hydrogels *in vivo* (Jastrzębska et al., 2017). However, GelMA still has the disadvantages of low absorptivity, inadequate mechanical strength, and limited antibacterial properties when used in wound repair. To address these shortcomings, researchers used different strategies to improve the performance of GelMA hydrogels by adding functional materials. In recent years, functional inorganic nanoparticles have attracted extensive attention because of their high efficiency in improving the tissue repair properties of biomaterials. These nanoparticles offer unique advantages such as enhanced mechanical strength, improved antibacterial activity, and enhanced bioactive molecule delivery, making them promising candidates for improving the functionality of GelMA hydrogels (Werner et al., 2007). MXene nanoparticles possess distinctive biological characteristics, making them excellent bio-loaded materials. The surface of MXene nanoparticles is rich in hydrophilic functional groups such as -OH, -O, and -F. This unique feature solves the complex surface modification problem required for hydrophobic nanoparticles. As a result, MXene nanoparticles offer expanded possibilities for biomedical applications, broadening their range of potential uses in the field. Zhou et al. prepared HPEM composite scaffolds with excellent rheological properties by adding MXene nanoparticles (Zheng et al., 2021). In this study, GelMA was used as the matrix material to prepare wound composite hydrogels by combining Os and MXene nanoparticles with purple linkage properties. By testing the effects of Os and MXene nanoparticles on the mechanical strength, degradation performance, swelling performance, and antibacterial performance of hydrogels, we found that GelMA/Os/MXene showed superior performance. Cell experiments showed that the GelMA material was further improved in biocompatibility and tissue repair ability after combination of the antibacterial peptide Os and MXene nanoparticles. Compared to other composite hydrogels, GelMA/Os/MXene composite hydrogels demonstrated increased cell numbers and enhanced cell proliferation activity. This can be attributed to the improved hydrophilicity and surface roughness resulting from the incorporation of Os and MXene, which provide additional cell binding sites. Moreover, the presence of antimicrobial peptides and abundant functional groups further enhances cell activity and inhibits bacterial growth. Notably, our research on rats revealed a synergistic effect between MXene and Os in wound healing. GelMA/Os/MXene nanocomposite hydrogel effectively accelerated wound healing, promoted epithelial remodeling, and facilitated collagen deposition. All in all, our study provides a new biomaterial and a new design concept for epithelial wound repair. In addition, it helps to have a deeper understanding of gelatin composite nanomaterials and provides a preliminary research basis for further preparation of wound healing materials in the future.

## Conclusion

In this study, we successfully encapsulated antibacterial peptides and MXene nanoparticles within a GelMA hydrogel composite. This hydrogel composite demonstrated good mechanical strength, swelling behavior, degradation properties, and antibacterial activity. Additionally, cell proliferation experiments confirmed that the composite hydrogels exhibited good biocompatibility. Finally, in a full-thickness mouse skin defect model, the prepared composite hydrogel was shown to effectively repair skin wounds.

In summary, the novel composite hydrogel system loading antimicrobial polypeptide Os and MXene nanoparticles possessed optimize properties for skin tissue regeneration, including sustained antibacterial efficacy, tunable mechanical support for wound closure, and promotion of cellular infiltration and tissue formation. These findings suggest potential applications of this biofunctionalized hydrogel platform for skin wound healing and regeneration. With further characterization of biochemical mechanisms *in vivo*, this composite may serve as an invaluable treatment option for infections and injuries of the skin. Overall, the results of this study validate the use of nanomodulated hydrogels for advancement of skin tissue engineering strategies.

## Data Availability

The original contributions presented in the study are included in the article/Supplementary Material, further inquiries can be directed to the corresponding author.
